# Ring-Oxidative Biotransformation and Drug Interactions of Propofol in the Livers of Rats

**DOI:** 10.1155/2015/658928

**Published:** 2015-02-01

**Authors:** Yu-Ting Tai, Yi-Ling Lin, Chia-Chen Chang, Yih-Giun Cherng, Ming-Jaw Don, Ruei-Ming Chen

**Affiliations:** ^1^Brain Disease Research Center, Department of Anesthesiology, Wan-Fang Hospital, Taipei Medical University, Taipei 116, Taiwan; ^2^Graduate Institute of Medical Sciences and Comprehensive Cancer Center of Taipei Medical University, Taipei Medical University, 250 Wu-Xing Street, Taipei 110, Taiwan; ^3^Department of Laboratory Medicine, China Medical University Hospital, Taichung 404, Taiwan; ^4^Department of Anesthesiology, Shuang-Ho Hospital, Taipei Medical University, Taipei 235, Taiwan; ^5^National Research Institute of Chinese Medicine, Taipei 112, Taiwan; ^6^Anesthetics Toxicology Research Center, Taipei Medical University Hospital, Taipei 110, Taiwan

## Abstract

Propofol, an intravenous anesthetic agent, is widely used for inducing and maintaining anesthesia during surgical procedures and for sedating intensive care unit patients. In the clinic, rapid elimination is one of the major advantages of propofol. Meanwhile, the biotransformation and drug interactions of propofol in rat livers are still little known. In this study, we evaluated the ring-oxidative metabolism of propofol in phenobarbital-treated rat livers and possible drug interactions. Administration of phenobarbital to male Wistar rats significantly increased levels of hepatic cytochrome P450 (CYP) 2B1/2 and microsomal pentoxyresorufin *O*-dealkylase (PROD) activity. Analyses by high-performance liquid chromatography and liquid chromatography mass spectroscopy revealed that propofol was metabolized by phenobarbital-treated rat liver microsomes into 4-hydroxypropofol. In comparison, PROD activity and 4-hydroxy-propofol production from propofol metabolism were suppressed by orphenodrine, an inhibitor of CYP2B1/2, and a polyclonal antibody against rat CYP2B1/2 protein. Furthermore, exposure of rats to propofol did not affect the basal or phenobarbital-enhanced levels of hepatic CYP2B1/2 protein. Meanwhile, propofol decreased the dealkylation of pentoxyresorufin by phenobarbital-treated rat liver microsomes in a concentration-dependent manner. Taken together, this study shows that rat hepatic CYP2B1/2 plays a critical role in the ring-oxidative metabolism of propofol into 4-hydroxypropofol, and this anesthetic agent can inhibit CYP2B1/2 activity without affecting protein synthesis.

## 1. Introduction

Propofol (2,6-diisopropylphenol) is an intravenous anaesthetic agent widely used to induce and maintain anaesthesia during surgical procedures [1, 2]. Propofol is also commonly applied to intensive care unit (ICU) patients as a sedative agent [3]. Our previous studies further showed the suppressive effects of propofol on downregulating inflammatory cytokine gene expression and reactive oxygen species production in gram-negative- and -positive-stimulated macrophages through toll-like receptor-dependent mechanisms [4–6]. Recently, we showed that propofol could protect cerebrovascular endothelial cells against nitrosative stress-induced apoptotic insults [7]. Furthermore, when applied in the clinic, propofol has the advantages of rapid onset, short action duration, and speedy elimination [8, 9]. A previous study reported that almost 80% of propofol was metabolically eliminated after male volunteers were intravenously infused with this anaesthetic agent for 30 min [10]. In humans, the liver is the major organ for the metabolic elimination of propofol [11].

The cytochrome P450 (CYP)-dependent monooxygenase system is a critical enzyme in the liver responsible for metabolism of a variety of endogenous and exogenous substrates, including drugs, carcinogens, hormones, and fatty acids [12, 13]. This monooxygenase system consists of phospholipids, NADPH-cytochrome P450 reductase, cytochrome *b*_5_, and a multiplicity of CYP isoforms. In human and canine livers, CYP2B isoforms are reported to participate in propofol metabolism [14–16]. However, the roles of rat hepatic CYPs in propofol metabolism are still little known. In addition, previous studies reported that propofol can cause drug interactions with certain CYP isoforms. For example, propofol was shown to decrease the clearance of midazolam and lidocaine possibly as a result of reducing hepatic CYP3A4 activity [17, 18]. In human CYP systems, propofol shows competitive inhibition to ropivacaine metabolism [19]. Our previous study exhibited that propofol possibly diminished kidney damage through suppressing renal CYP2E1 activity and enflurane metabolism to fluoride [20, 21]. Thus, the interaction of propofol with certain CYP members may alter the metabolism of this anaesthetic agent itself or other drugs.

CYP2Bs are a subfamily of proteins that contribute to the biotransformation of certain drugs and carcinogens such as cyclophosamide, cocaine, and aflatoxin B1 [22, 23]. In response to phenobarbital stimulation, hepatic CYP2Bs can be transcriptionally induced following cascade activation of a constitutively active receptor and the retinoid X receptor [24, 25]. The expression of CYP2B isoforms in the liver is species-specific. For example, the dominant members of hepatic CYP2Bs in rats, humans, and canines are 2B1/2, 2B6, and 2B11, respectively [14, 15, 25]. Rats are a popular animal model for evaluating the pharmacological and toxicological effects of a variety of drugs and toxicants [26]. However, which CYP isoforms in rat liver contribute to propofol biotransformation is little known. Therefore, in this study, we evaluated the roles of rat hepatic CYP2B1/2 in the ring-oxidative metabolism of propofol and possible drug interactions of this anaesthetic agent with this monooxygenase.

## 2. Materials and Methods

### 2.1. Animals and Drug Treatment

All of the protocols were approved by the Research Animal Care Committee of Wan-Fang Hospital, Taipei Medical University, Taipei, Taiwan. Briefly, male Wistar rats weighing 200~250 g were purchased from the Animal Center of the College of Medicine, National Taiwan University, Taipei, Taiwan. Before the experiments began, rats were allowed to acclimatize for at least 1 week in animal quarters with air conditioning and an automatically controlled photoperiod of 12 h of light daily. Animals were allowed free access to rodent laboratory chow (Purina Mills, St. Louis, MO, USA). Phenobarbital purchased from Sigma (St. Louis, MO, USA) was dissolved in distilled and deionized water. Propofol donated by Zeneca (Macclesfield, Cheshire, UK) was stored under nitrogen, protected from light, and freshly prepared by dissolving it in Intralipid. The purity of propofol was more than 98%. The Rats were intraperitoneally administered 80 mg phenobarbital or 80 mg propofol per day per kg body weight for 7 days. Control animals received water or Intralipid only.

### 2.2. Microsomal Preparation

After drug treatment, rats were sacrificed, and their livers were removed. After being washed, the livers were homogenized in ice-cold 1.15% potassium chloride. Microsomal fractions were prepared by differential centrifugation as described previously [27]. Liver microsomes were suspended in potassium phosphate buffer (100 mM, pH 7.4). Protein concentrations were determined using a bicinchoninic acid protein assay kit (Pierce, Rockford, IL, USA).

### 2.3. Assay of Pentoxyresorufin O-Dealkylase (PROD) Activity

PROD activity was assayed by measuring the fluorescence intensities of the dealkylated product, resorufin, as described previously [28]. In an* in vitro* enzyme inhibition study, orphenadrine, an inhibitor of CYP2B1/2 [29], at 12.5, 25, 50, and 100 *μ*M was incubated with liver microsomes and NADPH at 37°C for 10 min before pentoxyresorufin was added to the incubation mixture. The fluorescent products were detected using a fluorescence spectrophotometer (LS-55, PerkinElmer, Boston, MA, USA).

### 2.4. Immunoblotting Analyses of CYP2B1/2 and *β*-Actin

Protein analyses were carried out according to a previously described method [30]. To avoid degradation of the microsomal proteins by proteinases, a mixture of 1 mM phenyl methyl sulfonyl fluoride, 1 mM sodium orthovanadate, and 5 *μ*g/mL leupeptin was added to the radioimmunoprecipitation assay buffer. Proteins (50 *μ*g/well) were subjected to sodium dodecylsulfate polyacrylamide gel electrophoresis (SDS-PAGE) and transferred to nitrocellulose membranes. Levels of CYP2B1/2 in rat liver microsomes were immunodetected using a rabbit polyclonal antibody (pAb) against rat CYP2B1/2 (Chemicon, Temecula, CA, USA). *β*-Actin was immunodetected using a mouse monoclonal antibody (mAb) against mouse *β*-actin (Sigma, St. Louis, MO, USA) as the internal standards. These protein bands were quantified using a digital imaging system (UVtec, Cambridge, UK).

### 2.5. Assay of Propofol Metabolism

Ring-oxidative metabolism of propofol by liver microsomes was determined following the method of Court et al. [31]. Briefly, glass tubes were prepared on ice containing 50 *μ*L liver microsomes (100 *μ*g/mL protein) in 100 *μ*L of an NADPH-generation system (1 mM NADP, 3 mM MgCl_2_, 2 mM glucose-6-phosphate, 5 units/mL glucose 6-phosphate dehydrogenase, and 50 mM Tris-HCl; pH 7.5). The ring-oxidative reactions were initiated by adding propofol to the mixture and incubating it at 37°C for 10 min. Cyclohexane containing thymol as the internal standard was added to each tube to stop the oxidative reaction. After centrifugation, the organic layer containing propofol and its metabolites was dried under nitrogen gas. The extracted products were dissolved in a mobile phase for high-performance liquid chromatography (HPLC) analyses.

### 2.6. HPLC Analysis

Propofol and its metabolites were analyzed using HPLC as described previously [31]. The extracted products were dissolved in a mobile phase (60% acetonitrile in distilled water with 0.1% trifluoroacetic acid). The HPLC apparatus consisted of a controller with an autosampler (600S and 717plus; Waters, Milford, MA, USA) serially connected to an ultraviolet (UV) absorbance detector (PDA 996; Waters) or a fluorescence detector (474; Waters) depending on the assay method used. The UV detector was set to an absorbance wavelength of 270 nm, and the fluorescence detector was set to an excitation wavelength of 276 nm and an emission wavelength of 310 nm, and both monochromator slit widths were 10 nm. The column was a C18 reversed-phase column (XTerra RP_18_, 3.0 × 150 mm, 3.5 *μ*m; Waters). Data from HPLC were acquired and processed using the Millennium 32 software (Waters).

### 2.7. Analysis of Liquid Chromatography (LC) Mass Spectrometry (MS)

The LC-MS analysis was carried out as described previously [32]. The LC system (TSP SpectraSYSTEM, Brampton, Ontario, Canada) consisted of a quaternary pump (P4000; TSP), an autosampler (AS3000; TSP), and a UV detector (UV2000; TSP). Separation of propofol and its metabolites was performed using a C18 reversed-phase column (XTerra RP_18_; Waters) at ambient temperature. The mobile phase consisted of 60% acetonitrile, 40% water, and 0.1% trifluoroacetic acid with a flow rate 0.1 mL/min, and the absorbance at 270 nm was measured. A FinniganMAT LCQ ion trap mass spectrometer (ThermoQuest, San Jose, CA, USA) equipped with an electrospray ionization source was used. The mass spectrometric data were acquired in the positive ion full-scan mode from* m/z* 100–800 or selective ion monitoring mode at* m/z* 192.9 [M + H^+^] for 4-hydroxypropofol and at* m/z* 178.9 for propofol. Conditions for the electrospray mass spectra were as follows: a spray voltage of 4.5 kV; capillary voltage of 10 V; capillary temperature of 270°C; 80 units of sheath gas; and 20 units of auxiliary gas. Acquisition and processing of data from the mass spectrometer were performed using Xcalibur software revision 1.0 (ThermoQuest).

### 2.8. Assay of Chemical Inhibition

A chemical inhibition assay was analyzed following a previously described method [33]. Orphenadrine purchased from Sigma was dissolved in methanol. Various concentrations of orphenadrine were incubated with propofol and the microsomal enzyme-metabolizing system. The reaction was stopped, propofol was extracted by adding cyclohexame, and the extracted propofol and its metabolites were then dried under nitrogen gas. After dissolving the extracted products in the mobile phase, HPLC analyses were subsequently carried out.

### 2.9. Immunoinhibition Assay

An immunoinhibition assay was carried our according to a previous method [34]. Rat liver microsomes (20 *μ*g protein) were preincubated with a rabbit pAb against rat CYP2B1/2 (Chemicon) for 30 min at room temperature. The reaction was initiated by adding propofol and an NADPH cofactor solution. After the reaction, samples were extracted with cyclohexane and analyzed by HPLC.

### 2.10. Statistical Analysis

Statistically significant differences between the control and drug-treated groups were determined using Student's* t*-test, and differences were considered statistically significant at* P* values of <0.05. Statistical analysis between groups over time was carried out using two-way analysis of variance (ANOVA).

## 3. Results

### 3.1. Induction of CYP2B1/2 Protein and PROD Activity

**Figure 1:**
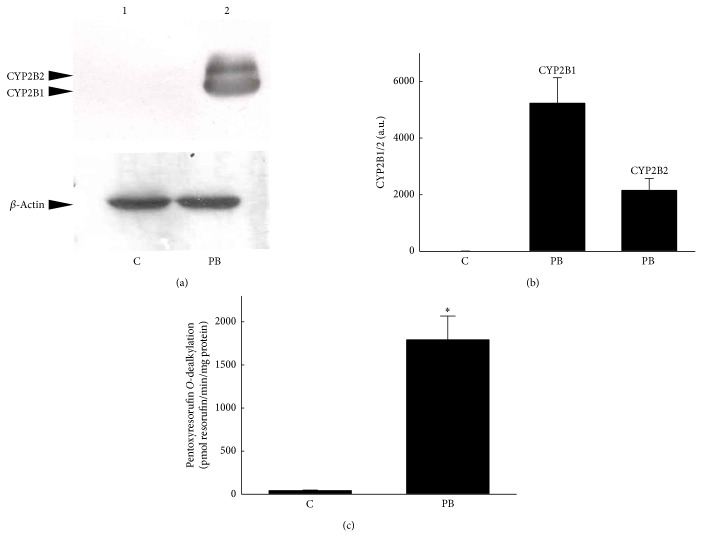
Effects of phenobarbital (PB) on CYP2B1/2 synthesis and enzyme activity. Rats were administrated 80 mg PB per kg body weight per day for 7 days. After drug treatment, animals were sacrificed, and the livers were removed, washed, and then homogenized for the microsomal preparation. Levels of CYP2B1/2 were immunodetected ((a),* top panel*). *β*-Actin was measured as the internal control (*bottom panel*). These immunorelated protein bands were quantified and analyzed (b). PROD activity was assayed by detecting the fluorescent product, resorufin (c). Each value represents the mean ± SD for *n* = 6. “^∗^” Values significantly differ from the respective control, *P* < 0.05. C, control.

An immunoblot analysis was conducted and enzyme activities were determined to evaluate the roles of CYP2B1/2 in the ring-oxidative metabolism of propofol (Figure 1). In untreated rats, the basal level of CYP2B1/2 in the liver was low (Figure 1(a),* top panel*, lane 1). Meanwhile, exposure of rats to phenobarbital obviously increased the amounts of CYP2B1/2 in liver microsomes (lane 2). Amounts of *β*-actin were immunodetected as the internal control (*bottom panel*). These immunorelated protein bands were quantified and analyzed (Figure 1(b)). Our results revealed that phenobarbital caused massive induction of hepatic CYP2B1/2. An assay of enzyme activities showed low PROD activity in control liver microsomes (Figure 1(c)). After exposure to phenobarbital, the PROD activity significantly increased by 34-fold.

### 3.2. Ring-Oxidative Metabolism of Propofol

**Figure 2:**
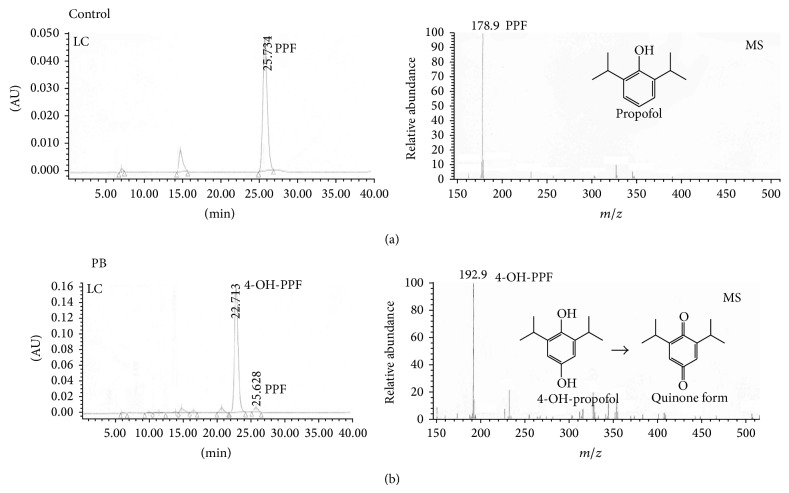
Identification of 4-hydroxypropofol. After reacting propofol with control or phenobarbital (PB)-treated liver microsomes in an NADPH-generation system, the metabolites were extracted with cyclohexane and dried with nitrogen gas. The extracted metabolites were dissolved in a mobile phase for analyses by liquid chromatography ((a), (b),* left panes*) and mass spectroscopy ((a), (b),* right panels*).

**Figure 3:**
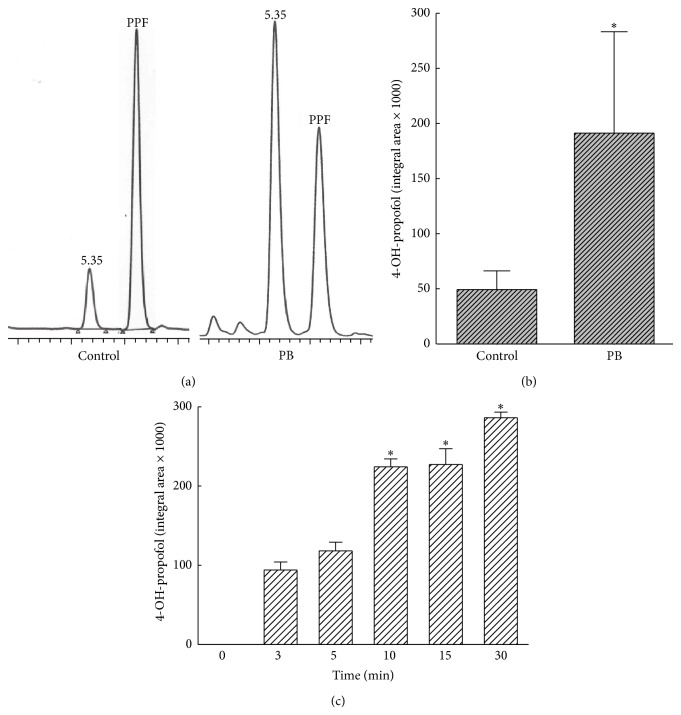
Ring-oxidative metabolism of propofol (PPF) into 4-hydroxypropofol. After reacting propofol with control or phenobarbital-treated liver microsomes in an NADPH-generation system, the metabolites were extracted with cyclohexane and dried with nitrogen gas. The extracted metabolites were dissolved in a mobile phase for the HPLC analyses (a). The peaks observed in the metabolism profile were quantified and analyzed (b). Time-dependent effects of propofol metabolism were determined (c). Each value represents the mean ± SD for *n* = 6. “^∗^” Values significantly differ from the respective control, *P* < 0.05.

Following metabolism by rat liver microsomes, propofol and its metabolites were analyzed using LC-MS and HPLC (Figures 2 and 3). Results of the LC analysis revealed that when propofol was metabolized by control rat liver microsomes, a major peak was observed at 25.7 min (Figure 2(a),* left panel*). The MS analysis further showed that this compound had the same fragment distribution as that of propofol (*right panel*). In comparison, after propofol was metabolized by phenobarbital-treated liver microsomes, in addition to propofol, a major peak was observed at 22.7 min (Figure 2(b),* left panel*). The MS analysis demonstrated that this compound was 4-hydroxypropofol (*right panel*).

The HPLC analysis showed that 4-hydroxypropofol appeared at 5.35 min (Figure 3(a)). After being metabolized by phenobarbital-treated liver microsomes, the amounts of 4-hydroxypropofol were greatly augmented (Figure 3(a)). Results of the HPLC analyses were quantified and analyzed (Figure 3(b)). The amounts of 4-hydroxypropofol were augmented by 4-fold in phenobarbital-treated liver microsomes compared to control ones. Metabolism of propofol into 4-hydroxypropofol by phenobarbital-treated rat liver microsomes was augmented in a time-dependent manner (Figure 3(c)).

### 3.3. Chemical Inhibition of PROD Activity and Propofol Metabolism

**Figure 4:**
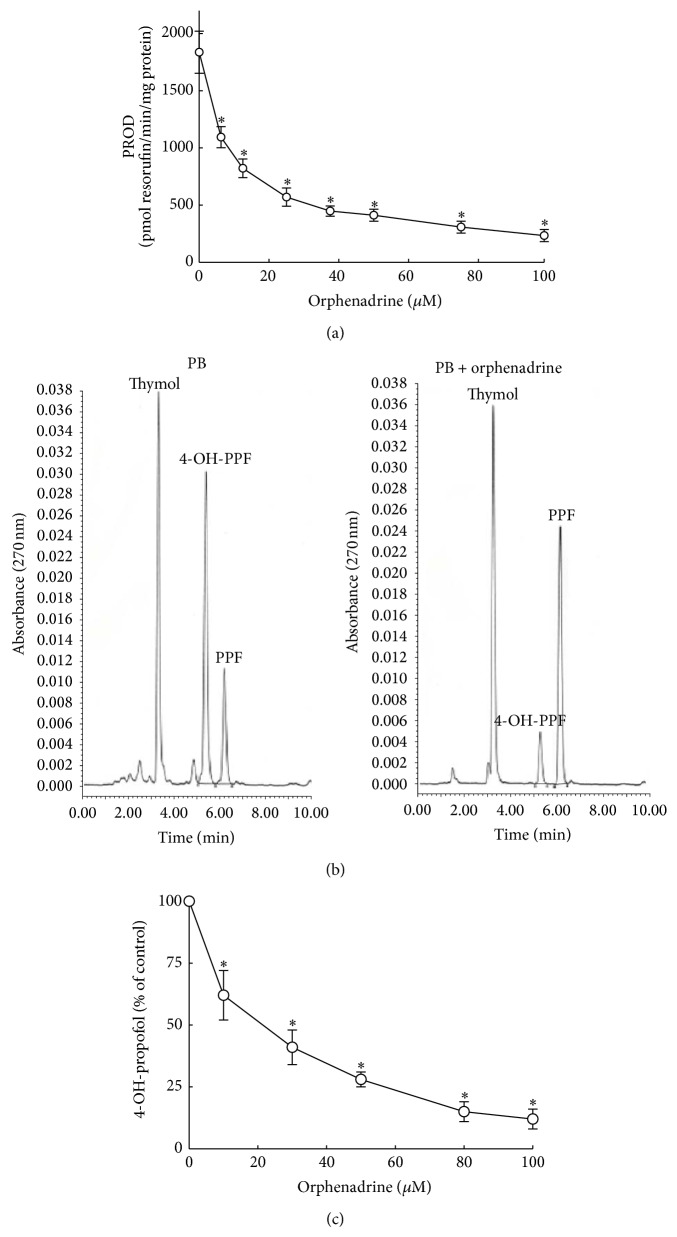
Roles of CYP2B1/2 in propofol hydroxylation verified by a chemical inhibition assay. Orphenadrine, an inhibitor of CYP2B1/2, was incubated with pentoxyresorufin or propofol. PROD activity was assayed by measuring the fluorescent product, resorufin (a). After reacting orphenadrine with propofol in phenobarbital (PB)-treated liver microsomes, the extracted metabolites were extracted, dried, and dissolved in the mobile phase for HPLC analyses (b). The concentration-dependent effects of orphenadrine on the metabolism of propofol into 4-hydroxypropofol were determined (c). Each value represents the mean ± SD for *n* = 6. “^∗^” Values significantly differ from the respective control, *P* < 0.05.

Orphenadrine, a specific inhibitor of CYP2B1/2, was used in this study to evaluate the role of CYP2B1/2 in propofol hydroxylation (Figure 4). Incubation with 6.25, 12.5, 25, 37.5, 50, 75, and 100 *μ*M orphenadrin, respectively, caused significant 38%, 56%, 60%, 76%, 78%, 84%, and 87% reductions in PROD activity (Figure 4(a)). After adding orphenadrine to the propofol metabolism reaction, the amounts of 4-hydroxypropofol metabolized by phenobarbital-treated liver microsomes obviously decreased (Figure 4(b)). These results were quantified and analyzed (Figure 4(c)). Orphenadrine at 10, 30, 50, 80, and 100 *μ*M significantly decreased the levels of 4-hydroxypropofol metabolized by 27%, 55%, 72%, 84%, and 87%, respectively (Figure 4(c)).

### 3.4. Immunoinhibition of PROD Activity and Propofol Metabolism

**Figure 5:**
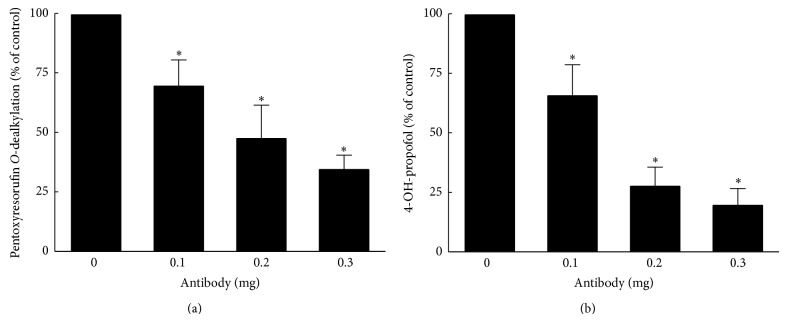
Roles of CYP2B1/2 in propofol hydroxylation verified by an immunoinhibition assay. A polyclonal antibody against CYP2B1/2 at 0.1, 0.2, and 0.3 mg was incubated with pentoxyresorufin or propofol. Pentoxyresorufin* O*-dealkylase (PROD) activity was assayed by measuring the fluorescent product, resorufin (a). After reacting 0.1, 0.2, and 0.3 mg of the CYP2B1/2 antibody with propofol in phenobarbital-treated liver microsomes, the extracted metabolites were extracted, dried, and dissolved in a mobile phase for the HPLC analyses. The HPLC results were quantified and statistically analyzed (b). Each value represents the mean ± SD for *n* = 6. “^∗^” Values significantly differ from the respective control, *P* < 0.05.

The roles of CYP2B1/2 in propofol ring-oxidation were further confirmed by an immunoinhibition assay (Figure 5). Application of a polyclonal antibody against CYP2B1/2 at 0.1 mg caused a 28% decrease in PROD activity (Figure 5(a)). Following application of 0.2 and 0.3 mg of the antibody, PROD activity, respectively, decreased by 51% and 65%. In parallel, when adding 0.1, 0.2, and 0.3 mg of the pAb against CYP2B1/2, levels of 4-hydropropofol dropped by 35%, 73%, and 80%, respectively (Figure 5(b)).

### 3.5. Effects of Propofol on CYP2B1/2 Synthesis and PROD Activity

**Figure 6:**
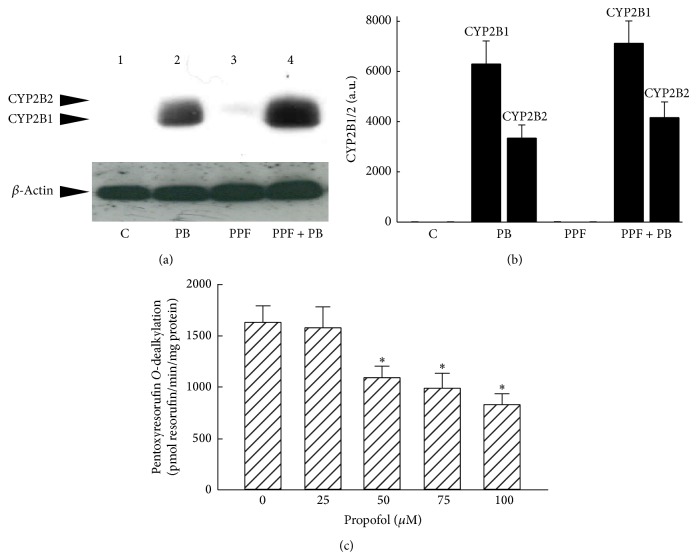
Effects of propofol on phenobarbital (PB)-induced CYP2B1/2 synthesis and pentoxyresorufin* O*-dealkylase (PROD) activity. Rats were exposed to propofol (PPF), phenobarbital (PB), and a combination of PPF and PB. Amounts of CYP2B1/2 were immunodetected ((a),* top panel*). *β*-Actin was quantified as the internal control (*bottom panel*). These immunorelated protein bands were quantified and analyzed (b). Propofol at 25, 50, 75, and 100 *μ*M was added for the dealkylation of pentoxyresorufin, and the enzyme activity in liver microsomes from control and PB-treated rats was determined by measuring the amounts of the fluorescent resorufin (c). Each value represents the mean ± SD for *n* = 6. “^∗^” Values significantly differ from the respective control, *P* < 0.05.

Drug interactions with propofol were evaluated by analyzing CYP2B1/2 synthesis and its specific PROD activity (Figure 6). Exposure of rats to phenobarbital obviously increased the amounts of CYP2B1/2 in liver microsomes (Figure 6(a),* top panel*, lane 2). Propofol alone did not affect hepatic CYP2B1/2 synthesis (lane 3). In comparison, treatment of rats with propofol did not influence phenobarbital-induced CYP2B1/2 expression (lane 4). Levels of *β*-actin were immunodetected as the internal control (*bottom panel*). These immunorelated protein bands were quantified and analyzed (Figure 6(b)). Cotreatment of rats with propofol and phenobarbital did not change the phenobarbital-induced CYP2B1/2 expression by liver microsomes. Analyses of enzyme activities revealed that propofol at 25 *μ*M did not affect PROD activity in liver microsomes from phenobarbital-treated rats (Figure 6(c)).

## 4. Discussion

CYP2B1/2 plays a critical role in the ring-oxidative metabolism of propofol in rat livers. This study showed that exposure of rats to phenobarbital increased CYP2B1/2 expression and its specific enzyme activities. In parallel, analyses by HPLC and LC-MS demonstrated the ring-oxidative metabolism of propofol into 4-hydroxypropofol by phenobarbital-treated microsomes. In comparison, when CYP2B1/2 activity was reduced by orphenadrine and its specific antibody, 4-hydroxypropofol production was concurrently inhibited. Thus, the results presented in this study show that rat hepatic CYP2B1/2 contributes to the ring-oxidative metabolism of propofol into 4-hydroxypropofol. Propofol can be used to induce anaesthesia during surgical procedures and sedate ICU patients [1–3]. When applied in the clinic, propofol has the advantage of rapid elimination through being biotransformed into more-hydrophilic metabolites in the liver [8, 9]. Simons et al. reported that almost 80% of propofol was metabolically eliminated in male volunteers within 30 min [10]. As a result, this study provides* in vivo* data to demonstrate the metabolic elimination of propofol in rat livers mainly due to the contribution of CYP2B1/2.

The CYP2Bs responsible for propofol hydroxylation are species-specific. An immunoblot analysis revealed that levels of CYP2B1/2 in rat livers were greatly enhanced after exposure to phenobarbital. Previous studies disclosed that phenobarbital is a good stimulator for inducing CYP2Bs [23, 24]. The present results also illustrate increased PROD activity in phenobarbital-treated liver microsomes. PROD is characterized by CYP2B1/2-specific enzyme activity [35]. Previous studies reported that the dominant members of hepatic CYP2B in rats, humans, and canines are 2B1/2, 2B6, and 2B11, respectively [14, 15, 25]. Thus, phenobarbital can exclusively induce CYP2B1/2 protein expression and its enzyme activities. This study showed that propofol was metabolized into 4-hydroxypropofol by phenobarbital-treated liver microsomes, and this biotransformation was suppressed by orphenadrine and a specific antibody. Orphenadrine is an inhibitor of CYP2Bs [29]. In the human liver, CYP2B6 is reported to be a major enzyme that participates in hydroxylation metabolism of propofol [14, 15]. By comparison, CYP2B11 plays a crucial role in propofol hydroxylation in canine liver microsomes [16]. In contrast, this study further showed that CYP2B1/2 is a key enzyme in the ring-oxidative metabolism of propofol in rat livers.

4-Hydroxypropofol is a major metabolite in CYP2B1/2-involved monooxygenation of propofol in rat livers. The HPLC analysis revealed that after propofol was biotransformed by phenobarbital-treated rat liver microsomes, a major metabolite was observed in the profile. The major metabolite was more hydrophilic than the parent compound, propofol. Results of the LC-MS analyses further showed that this metabolite was a hydroxylated product. Court et al. reported that hydroxypropofol can automatically be oxidized into the relative quinine form [31]. Accordingly, the hydroxylated quinine metabolite measured in the HPLC profile of this study was obtained from 4-hydroxypropofol. Previous studies presented similar results of the production of 4-hydroxypropofol representing a significant pathway in propofol metabolism in human and dog livers [14, 31]. In addition, Acco and Bracht stated that the liver functions as a 4-hydroxypropofol source for conjugation to glucuronic acid or sulfate in other tissues [36]. For that reason, 4-hydroxypropofol is a key ring-oxidative metabolite in rat livers, and it might be transported elsewhere for further glucuronide conjugation and consequent elimination.

Propofol causes drug interactions with hepatic CYP2B1/2. After exposure to propofol, the basal and phenobarbital-enhanced levels of CYP2B1/2 in rat livers were not altered. Meanwhile, coincubation of propofol led to a significant reduction in CYP2B1/2-specific PROD activity. A previous study showed that when propofol was intravenously administered to rats, CYP2B-involved testosterone 16beta-hydroxylation and pentoxyresorufin depentylation in rat liver microsomes decreased [37]. Administration of propofol also induces strong suppression of CYP2C functions. In human liver microsomes, propofol can reduce CYP3A4 activity and then competitively inhibit ropivacaine metabolism [19]. Gemayel et al. revealed the suppressive effects of propofol on hepatic CYP1A2 and CYP2B1 activities in their* in vitro* and* in vivo* studies [38]. In addition, our previous studies showed that clinically relevant concentrations of propofol exhibit broad-spectrum inhibition of hepatic and renal monooxygenase activities and enflurane defluorination [20, 21]. Those studies revealed the occurrence of drug interactions during propofol administration. The present study further showed the suppression of PROD activity, characterized by CYP2B1/2-specific enzyme activity [35], in rat liver microsomes by propofol. CYP2B1/2 contributes to the biotransformation of a variety of drugs and carcinogens, including cyclophosamide, cocaine, and aflatoxin B1 [22, 23]. Thus, our present results suggest that the propofol-induced deactivation of CYP2B1/2 has important implications for drug metabolism by rat liver microsomes.

In the human liver, multiple CYP isoforms, including CYP2A6, -2B6, -2C8, -2C18, -2C19, and -1A2, are reported to participate in propofol metabolism [14–17]. In canine liver microsomes, CYP2B11 was shown to play a critical role in propofol hydroxylation [16]. This study provides several lines of evidence, including enzyme induction, chemical inhibition, and immunoinhibition, to illustrate the roles of CYP2B1/2 in propofol hydroxylation. In the clinic, propofol can be used to induce and maintain anaesthesia during surgery and to sedate ICU patients [1–3]. The liver is the major organ for the metabolic elimination of propofol [11]. Rats are a popular animal model used to investigate the pharmacological and toxicological characteristics of drugs and toxicants [26]. As a result, elucidating the mechanism of propofol metabolism in the rat liver may be beneficial for future studies of propofol when rats are used as an experimental model. However, there are certain study limitations in this study, including (1) the roles of the other enzyme systems such as CYP2C11 and glucuronide-conjugated Phase II enzymes in propofol metabolism are not elucidated and (2) we cannot rule out if propofol metabolites contribute to inhibition of PROD activity. We will continue to investigate these critical issues in our upcoming study.

## 5. Conclusions

In summary, the present study shows that administration of phenobarbital to rats increased CYP2B1/2 protein synthesis and enzyme activities. HPLC and LC-MS analyses disclosed that propofol was metabolized into 4-hydroxypropofol by phenobarbital-treated liver microsomes. A chemical inhibition assay demonstrated the simultaneous reduction in PROD activity and propofol hydroxylation. The role of CYP2B1/2 in the biotransformation of propofol into 4-hydroxypropofol was alternatively confirmed by an immunoinhibition assay. Exposure of rats to propofol did not influence basal or phenobarbital-augmented levels of CYP2B1/2 in liver microsomes. However, propofol exhibited competitive inhibition against PROD activity. Taken together, this study demonstrates the roles of CYP2B1/2 in the ring-oxidative metabolism of propofol into 4-hydroxypropofol in rat livers and drug interactions of this intravenous anaesthetic agent with this monooxygenase.
